# Selections

**Published:** 1892-12

**Authors:** 


### Selections.

### DENTITION IN INFANTS.

A communication from the pen of H. C. Wood, upholding gum-lancing, and taking issue with the views of Forchheimer on the subject, as detailed in his recent book on the "Diseases of the Mouth," has been copied very generally by the medical press of the country. This extensive republication may fairly be taken as expressive of a general approval of the position taken by Professor Wood.

Upon the questions of difficult dentition and gum-lancing, the medical world has been for some years divided, the smaller party taking the modern view that dentition is a normal process, and rarely, if ever, produces dangerous symptoms, the larger party holding that dentition is responsible for most of the ills that infants suffer from, and that gum-lancing is its sovereign remedy. This latter view is one of our most ancient possessions, having come down to us from Hippocrates. For centuries it remained unquestioned, and has consequently become firmly intrenched in both the professional and the lay mind.

During the past summer this subject has occupied somewhat the attention of the Academy of Medicine of Paris. At the meeting of July 12, Magitot said, "We wish that the so-called diseases of dentition might be definitely erased from our medical nosology."

This brought a reply from M. Pamard on August 9, who took the following ground:

1. All difficult dentition is accompanied by a disturbance of the health of the infant.

2. In cold climates and in cold seasons all difficult dentition is accompanied by reflex phenomena on the part of the respiratory organs. In warm climates and in warm seasons all difficult dentition is accompanied by reflex phenomena on the part of the digestive organs.

3. The diseases allied to dentition in the infant pursue a course and present characteristics which are clearly defined and well established.

These propositions were supported only by the old argument of coincidence, but the essayist was upheld by MM. Le Roy de Mericourt, Herard, Charpentier, Peter, and Constantin Paul. He was opposed by MM. Ollivier and Hardy.

In the study of this question it is first necessary to separate dentition and gum-lancing. The first is a possible pathological condition, while the second is a therapeutic procedure.

*We think it can be said without fear of contradiction that there is not a single positive observation which has ever been recorded to prove that dentition produces general or reflex symptoms.* [Italics ours.] It is undeniable that at the period in life when dentition is in progress the infant is subject to certain disorders which occur much more commonly than at any other period of life.

If it could be shown that dentition was the only peculiarity of the infant, then its causative influence would be clear. But dentition is not the only peculiarity of the infant, and co-existing phenomena can only be classed as coincident. The most profound characteristic of infancy is that it is the period of most rapid growth and development of all organs; and careful observation of infants reveals numerous and great deviations from the normal growth and development in many instances. It will probably not be denied that such deviations are found most commonly in infants who have been artificially fed. In infants improperly fed, and this term is too extensive to attempt to define here, reflex manifestations are very readily produced, and it is not improbable that even a normally developing tooth may, in such an infant, be the exciting cause of trouble. We have seen infants who would invariably have a bronchial attack immediately before the proruption of a tooth, but they have invariably been infants who were suffering from demonstrable deviations from normal nutrition. We have further found that after improving the nutrition of these infants, the further progress of dentition was unaccompanied by symptoms.

In such cases, while it would be just as well, perhaps, to recognize the possible influence of dentition, its subordinate importance should be kept clearly in view. The great danger of teething is in the diagnosis, for when this is once made, the important underlying conditions are apt to be neglected, and permitted to progress to the death of the child.

But if dentition cannot be shown to be the great etiological factor of infantile disorders, it does not follow that gum-lancing should be abandoned. It is difficult to overlook the numerous instances in which careful observers have thought they have obtained good results from its use, but it would be well also to bear in mind the many cases in which it has failed. As a therapeutic procedure, it may have some value, but the indications for its use must be sought elsewhere than in a supposititious condition of teething. We would like to offer the following conclusions:

1. Before the diagnosis of "teething" is made, there should first be carefully excluded organic disease of all organs,—infection, intoxication, and perversion of nutrition.

2. Gum-lancing as a therapeutic measure should stand on its own merits, and be studied apart from any supposititious and undemonstrable process of teething.—*Abstracts from Editorial in Journal of the American Medical Association.*

### PROFESSOR HARRIS.

The following abstracts from a letter in relation to some facts connected with Professor Harris, of Baltimore, will, no doubt, be interesting to those who look upon Dr. Harris as the father of modern dentistry. The letter is written by a daughter, Mrs. C. G. Burrett Lennard, of London. She says, speaking of the portraits extant of her father:

"We have several photographs, two of which my mother and sisters considered good, and there have been a good many portraits painted, but the one by Mr. Eastman Johnson, of New York, is, I imagine, the only one of artistic merit. It was, unfortunately, prepared after his death from photographs, aided, no doubt, by memory, as Mr. Johnson was an old friend. . . . It is like him, but conveys no idea of the man, but is rather that of a 'country squire,' if that English term conveys any impression. It looks like a handsome, hale, jolly sort of person, without any special mark of intellectual strength and spirituality. . . . My father was remarkably handsome and intellectual looking. . . . He was six feet two and a half inches, broad-shouldered, and finely proportioned. . . .

"At an early age my father was deeply impressed by a sermon at a Methodist Episcopal church, and was induced to join it. . . . He was a deeply religious man, very benevolent, and given to all sorts of good works. He was very large-minded, and so free from having sectarian bias, he numbered among his intimate friends Romish priests, Episcopalian clergymen, and men attached to various Protestant bodies. During his last illness they all visited him constantly, and once five of them, including a Romish priest, and the present bishop of Western New York, all chanced to meet on his door-step. He was especially fond of the Episcopal church, and was in the habit of going to it often. . . . My father died on Michaelmas-day, September 29, 1860. . . .

"My father was the youngest of three brothers, both of whom died long before him, and whose families are passed away. . . . My youngest uncle had two children,—a daughter . . . in Minnesota and a son in Kentucky. . . . These are all the relations on my father's side, except some cousins, daughters of his eldest sister."

Mrs. Lennard then denies that any one in Baltimore in the practice of dentistry can "claim relationship with us, . . . as they are not even *distantly* connected."

### JAWS OF INEBRIATES.

Having thus found the cause, the question naturally arises, "Shall we not find as large a percentage defective among inebriates as other neurotics?" I have therefore examined the mouths of all of these classes. I will now give you the results of my investigations, taking them in the order in which they were made.

TABLE OF DEFORMITIES OF THE JAWS OF THE DEGENERATES.

| | No. | Normal. | Large Jaw. | High Vault. | V-Shaped Arch. | Partial V-Shaped Arch. | Semi-V-Shaped Arch. | Saddle Arch. | Partial Saddle Arch. | Semi-Saddle Arch. |
|---|---|---|---|---|---|---|---|---|---|---|
| Idiots. . . . . . . . . . . | 1977 | 55.3 | 7.6 | 16. | 6.5 | 11.9 | . . . | 10.4 | . . . | . . . |
| Deaf and Dumb . . . . . | 1935 | 45.3 | 15.7 | 21.7 | 8.7 | 9.9 | . . . | 10.4 | . . . | . . . |
| Blind. . . . . . . . . . . | 207 | 50.7 | 7.7 | 18.3 | 3.3 | 4.3 | . . . | 5 3 | . . . | . . . |
| Insane . . . . . . . . . . | 700 | 62. | 18. | 44. | 26. | 47. | . . . | 12. | . . . | . . . |
| Criminal . . . . . . . . . | 477 | 36.06 | 15.72 | 14.67 | 2.7 | 16.56 | 3.9 | 12.36 | 19.28 | 5.03 |
| Inebriates* . . . . . . . . | 514 | 25.04 | 6.4 | 59.5 | 1.5 | 24.4 | 0.3 | 9.3 | 18.2 | 7.7 |
| Normal. . . . . . . . . . | 1000 | 78. | 1.9 | 5.6 | 1.1 | 6.1 | . . . | 3.3 | . . . | . . . |

* The examination of inebriates was made in the Keeley Institute, Dwight, Ill.; the Inebriates Home, Ft. Hamilton, N. Y.; Washingtonian Home, Chicago; Washingtonian Home, Boston; and Dr. Crothers's Institute, Hartford, Conn.

It will be observed that there is a larger percentage of deformities among the inebriates than among any other defective class.

These deformities, however, are not so *pronounced* as those found among the idiots and criminal classes. The large percentage of deformities and high vaults indicate a strong neurotic tendency early in life, even before the seventh year. Finally, if the child from the seventh to the twelfth year has arrested development of the superior maxilla, and possesses constitutional irregularities of the teeth and a high vault, we can almost invariably say that the individual will become, not always an idiot, but a genius in some one direction or an idiot, deaf, dumb, blind, insane, a criminal, an extreme egotist, excessive tobacco user, or an inebriate,—all of these conditions coming under the head of degenerates, or neurotics. In making a diagnosis of any one of these classes of individuals mentioned, we should always take into account these three conditions of deformities of the jaws,—viz., arrest of development of the superior maxilla, constitutional irregularities of the teeth, and high vaults.—Dr. Eugene S. Talbot, *Journal of the American Medical Association.*

### DEATH UNDER PENTAL.

THE death of a German opera-singer is reported by the *British Medical Journal*, July 9, 1892, to have recently occurred after the administration of pental for the extraction of a tooth. Eight or ten drops were given upon a face-piece and the tooth removed, whereupon the patient fell back with a loud shriek and never recovered consciousness, attempts to restore animation proving of no avail. The official examination showed that the brain, lungs, and vessels were healthy; there was, however, some amount of fatty degeneration of the heart, and death was attributed to syncope, probably due to the condition of that organ.—*New York Medical Record.*

---

### EXAMINATION OF SOME COMMERCIAL VARIETIES OF HYDROGEN DIOXIDE.

BY HENRY LEFFMANN, M.D., AND WILLIAM BEAM, M.D., PHILADELPHIA.

HYDROGEN dioxide, generally inaptly called hydrogen peroxide, has found, in recent years, many valuable applications in preventive medicine and therapeutics. The pure article being very difficult to prepare and preserve, the commercial forms consist of dilute solutions in water, containing a very small amount of actual dioxide. Since the medicinal value of the article depends on its strength and freedom from impurities, it is important to make some comparative determinations on these points of the brands of hydrogen dioxide now offered in the market. The tests employed have been frequently described, and it will not be necessary to repeat them here. Among the impurities, free acid is the most objectionable, on account of its irritating action.

Comparative tests of commercial brands have from time to time been published in medical and pharmaceutical journals, but usually without indication of the identity of the samples, and, therefore, of but little practical value. In judging of the strength of any sample, the unavoidable liability to decomposition must be regarded. On the other hand, the proportion of free acid and of metallic salts will not be appreciably affected by the lapse of time.

In the following examination care was taken to secure fresh samples, and they were all in the original, unbroken packages when delivered to us. The proportion of hydrogen dioxide is expressed, as is customary, in volumes of oxygen, determined by the standard method with potassium permanganate:

| Brand. | Volumes of oxygen. | Barium salts. | Earthy salts other than barium. | Acidity expressed in milligrammes of KHO required for 25 c.c. of sample. |
|---|---|---|---|---|
| O. C. (Oakland Chemical Company) | 15.6 | None. | None. | 3.8 |
| Béné . . . . . . . . . . . . . . . | 11.8 | " | " | 3.36 |
| Lehn & Fink . . . . . . . . . . . | 10.5 | " | " | 4.08 |
| Marchand (first sample) . . . . . | 10.1 | " | Present. | 34.08 |
| " (second sample)* . . . . | Not determined. | " | None. | 22. |
| Powers & Weightman . . . . . . | 9.02 | " | " | 23.04 |
| Merck . . . . . . . . . . . . . . | 12.09 | " | Large amount. | 16.32 |
| Mallinckrodt . . . . . . . . . . | 9.14 | " | Traces. | 8.64 |

* This sample was one that had been purchased from some laboratory experiments in bleaching, and having been kept without any precautions to preserve its strength, the determination of the volume of O would have been of no value in indicating the original quality; but since the amount of free acid and earthy salts will not be affected by such treatment, they were determined. The volume strength claimed for the sample originally was less than that claimed for sample No. 1.

—*Medical News.*

### CHEMISTRY OF THE CHOLERA BACILLUS.

At a recent meeting of the French Academy of Sciences, attention was directed by M. Ferran to the fact that the *Bacillus mastitidis* (Guillebeaux), the *streptococcus mastitidis sporadicæ*, the *streptococcus scarlatinæ*, the *Bacillus diphtheriæ*, the *Bacillus coli communis*, the *Bacillus ovale ilei*, the *Bacillus Gaffky*, and the *Bacillus Schardingerii* determine the fermentation of milk, and, by their action on the lactose, the production of paralactic acid, with this peculiarity: that the acid produced by them is dextro-rotary, while that produced by others is lævo-rotary. When the comma bacillus is cultivated in a slightly alkaline bouillon, containing lactose, paralactic acid is produced in quantity sufficient to render the liquid distinctly acid. When this microbe is sown upon agar that is slightly alkaline, and containing lactose as well as litmus, the medium becomes red from the production of paralactic acid. A cultivation in a slightly alkaline bouillon, containing lactose, presents, after being left at rest at 30° C. for five days, a floating mycoderm, consisting of large comma bacilli, in the interior of which may be seen one or two very small granulations analogous to spores; eventually all the protoplasm of the bacillus disappears, leaving exposed these small granulations, which are readily colored by methyl-violet. The same comma bacillus of cholera sown in a small quantity of alkaline bouillon, contained in a capacious flask, may remain alive for more than three years, provided that the flask is closed by cotton-wool, which will allow of the renewal of the air. Under the very same conditions, and with the sole difference that the bouillon contains some lactose, the vitality of the microphyte is rapidly extinguished by reason of the acid character communicated to the medium by its own action. In ordinary culture bouillons the growth of this microbe is always rapid and luxuriant; but when the bouillon contains lactose it is disproportionately less prolific. The colonies become very numerous in consequence of the addition of that substance within a few hours; but the growth ceases completely as soon as the medium becomes acid, and before long the vitality of the microbe is destroyed. M. Ferran remarks that it is for medical men to deduce from these facts whatever rational indications they may contain in regard to the treatment of cholera. His present object is to call attention to the resemblances which obtain between the chemical function of this microbe and that of *Bacillus coli communis.* In many particulars their pathogenic functions are also similar. Paralactic acid paralyzes the chemical activity of both. The known value of that acid as a remedy for the diarrhœa caused by *Bacillus coli* suggests the probability that its administration in cases of diarrhœa caused by the comma bacillus might be equally efficacious and beneficial. M. Ferran therefore thinks that as a remedy for cholera it would be reasonable to administer lactic acid in the form of lemonade, and to assist its action by the anexosmotic power that is offered by morphine, which might probably prevent the absorption of toxines and prolong the influence of the lactic acid by reducing the rapidity of its elimination.—*Pharmaceutical Journal.*

